# S‐Methyl Methanethiosulfonate, the Main Human Metabolite of S‐Methyl‐L‐Cysteine Sulfoxide, Alters Energy Metabolism in Prostate Cancer Cells

**DOI:** 10.1002/mnfr.70008

**Published:** 2025-03-09

**Authors:** Gemma Beasy, Federico Bernuzzi, Priscilla Day‐Walsh, Perla Tronsco‐Rey, Marianne Defernez, Shikha Saha, Richard F. Mithen, Maria H. Traka, Paul A. Kroon

**Affiliations:** ^1^ Quadram Institute Bioscience Norwich UK; ^2^ Cancer Research UK Scotland Institute Garscube Estate Glasgow UK; ^3^ University of Glasgow School of Cancer Sciences Wolfson Wohl Cancer Research Centre Garscube Estate Glasgow UK; ^4^ Centre for Trophoblast Research Department of Obstetrics and Gynaecology University of Cambridge Cambridge UK; ^5^ University of East Anglia Norwich UK; ^6^ Liggins Institute University of Auckland Auckland New Zealand

**Keywords:** broccoli, DU145 prostate cancer cells, energy metabolism, MMTSO, SMCSO

## Abstract

Cruciferous and allium vegetables contain the sulfur compound S‐methyl‐L‐cysteine‐sulfoxide (SMCSO). Considering SMCSO is found at a higher abundance compared to the glucosinolates, there are limited reports on its effect on health, with the majority of the evidence on the beneficial effects on glucose metabolism in rodent models. In the current study, we investigated the metabolic effects of SMCSO and its metabolite, S‐methyl methanethiosulfonate (MMTSO), on prostate cancer metabolism. DU145 prostate cancer cells were cultured in 5.5 mM (basal), 10 mM (intermediate) and 25 mM (high) glucose concentrations in the presence of SMCSO or MMTSO (100 µM). Using Seahorse technology, MMTSO but not SMCSO reduced mitochondrial metabolism, mitochondrial ATP, and the percentage of oxidative phosphorylation and increased the fatty acid dependency of DU145 cells. Transcriptomic and metabolomic analyses observed cellular and energy metabolism pathways and immune response changes.

These data show that MMTSO alters several features of energy metabolism in DU145 prostate cancer cells, shifting them towards a non‐cancerous phenotype. These data are consistent with the notion that MMTSO may contribute to the beneficial effects of a broccoli‐rich diet and metabolic effects of prostate cancer.

## Graphical abstract

In this study, we show that the sulfur compound commonly found in cruciferous and allium vegetables and the main human metabolite of S‐methyl‐L‐cysteine sulfoxide, called S‐methyl methanethiosulfonate (MMTSO), alters energy metabolism in DU145 prostate cancer cells. MMTSO may contribute to the beneficial effects of a broccoli‐rich diet and metabolic effects of prostate cancer.

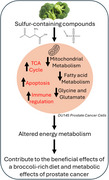

## 1 Introduction

**FIGURE 1:**
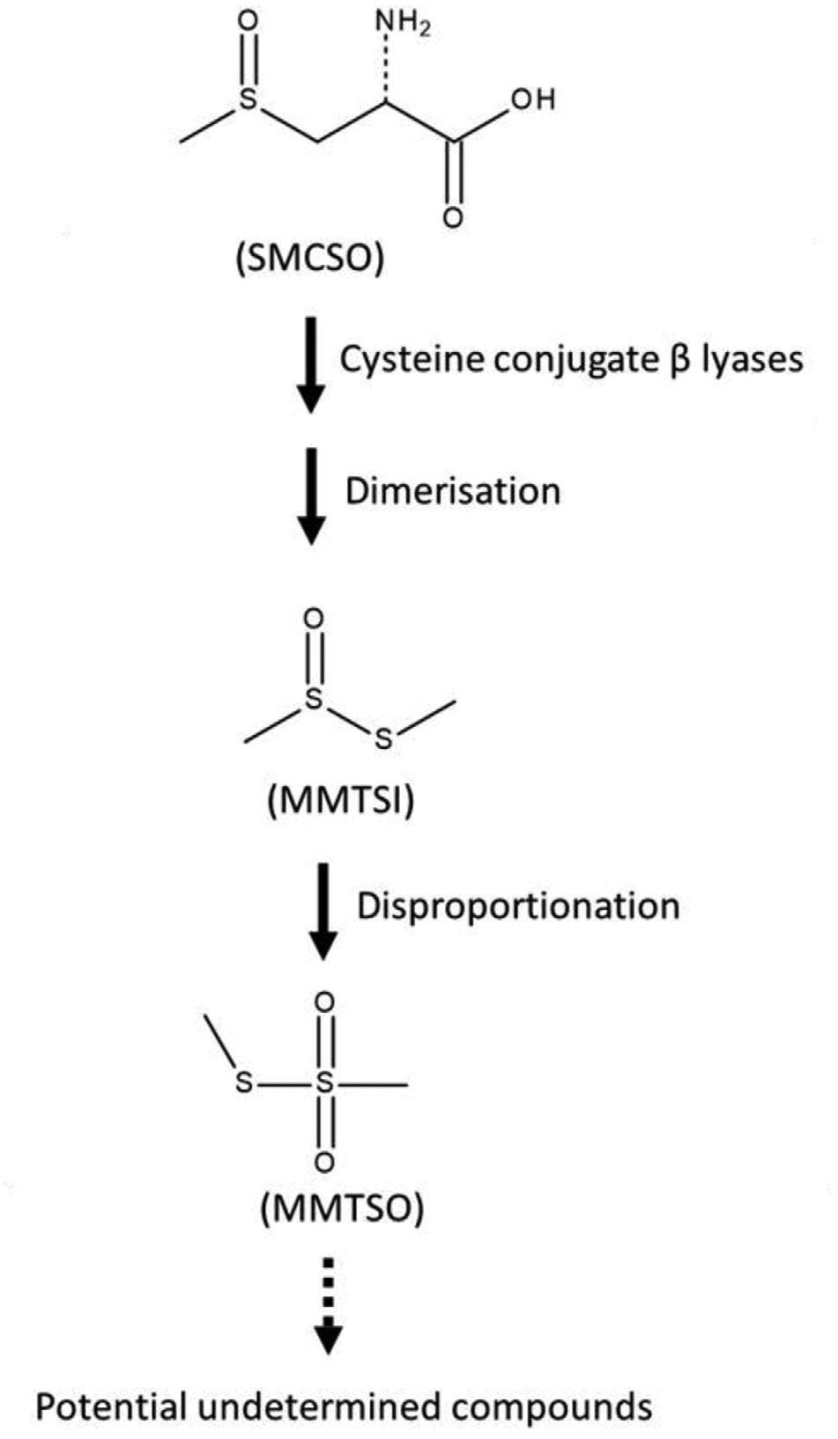
Metabolism of S‐methyl‐L‐cysteine sulfoxide (SMCSO). Initially, SMCSO is broken down by cysteine conjugate β lyases and then through dimerization to generate S‐methyl methanethiosulfinate (MMTSI). Further disproportionation leads to the generation of S‐methyl methanethiosulfonate (MMTSO) and potential undetermined compounds. Adapted from Livingstone et al. [28] and Beasy et al. [23]

Cruciferous vegetables such as broccoli contain sulfur compounds. These sulfur compounds have been reported in epidemiological studies to have health benefits across multiple conditions, including cancer [1, 2, 3] and diabetes [4, 5]. Examples of these sulfur compounds include glucoraphanin and S‐methyl‐L‐cysteine sulfoxide (SMCSO). SMCSO is often present in greater concentrations (1%–4% dry weight) in comparison to glucosinolates (typically 0.1%–0.6% dry weight) in Brassica [6]. SMCSO is converted to the metabolite S‐methyl methanethiosulfonate (MMTSO; also called methyl methanethiosulfonate or dimethyl disulfide sulfone) by microbial or plant cysteine lyase enzyme (Figure 1). The mechanism for SMCSO metabolism and further metabolism of MMTSO in humans remains unclear, although gut microbiota has been reported to play a role [7, 8].

Considering that SMCSO is widely distributed across commonly consumed plant foods (cruciferous and allium vegetables) and is present at a higher abundance compared to the glucosinolates [9], there are surprisingly few reports concerning the potential effects of SMCSO and MMTSO on health. Very few in vitro studies have investigated the impact of these compounds. In one report, the treatment of prostate cancer cell lines with SMCSO did not change mitochondrial or glycolytic function, and neither were there significant changes in reactive oxygen species (ROS) or the redox status of the phosphatase and tensin homolog (PTEN) protein in normal prostate epithelial cells [10]. Another study demonstrated that exposure of hepatoma cells (HepG2) to MMTSO and S‐methyl methanethiosulfinate (MMTSI) decreased mitochondrial potential, upregulated pathways relating to immune response, including IL‐6/JAK/STAT3 signaling, and downregulated cancer‐related pathways, including angiogenesis and KRAS signaling [11]. Limited in vivo studies have reported the anticarcinogenic effects for MMTSI and MMTSO [12, 13], potentially due to the presence of thio‐sulfinate and thiol functional groups [9, 14]. There have been only two reports of human studies with SMCSO: one investigating the excretion [15] and the other its accumulation in prostate tissue [16]. While reports show that supplementation of diets with SMCSO causes reductions in fasting plasma glucose in rodent models [17, 18, 19], there are no studies of the potential impact of SMCSO on human health and disease. Thus, SMCSO and/or MMTSO may have unidentified biological properties and may play a role in the protective effect of a broccoli‐rich diet.

SMCSO has been reported to accumulate in the human prostate [16] and also identified in the urine and plasma of individuals who have consumed broccoli soups [16, 20]. Potentially direct exposure to the prostate may have a biological effect, but urinary reflux and/or systemic exposure could also play a role. Thus, investigating the effect of direct exposure to SMCSO metabolites such as MMTSO on prostate cancer cells is key for understanding the metabolic effects of these compounds on the cancer cells.

In this study, we aimed to investigate the effects of SMCSO and its metabolite, MMTSO, on prostate cancer metabolism. We used DU145 cells, as this cell line represents an established prostate cancer cell line that has only moderate metastatic potential, and this was relevant to our research into chemoprevention. While PC3 is also a well‐established and characterized prostate cancer cell, PC3 cells have high metastatic potential and are therefore a cell model for a more advanced stage of prostate cancer [21]. We exposed these cells to varying concentrations of SMCSO and MMTSO under different glucose conditions. In addition to basal glucose (5.5 mM), intermediate (10 mM) and high (25 mM) glucose environments were also used, as these have been reported to increase cell proliferation, promote inflammation, and induce oxidative stress [22]. We assessed the metabolic effects of SMCSO and MMTSO on mitochondrial function and their impact on prostate cell transcript and metabolite profiles [23].

## 2 Experimental Section

*Cell culture*: Human prostate adenocarcinoma cell line DU145 (Cat. HTB‐81) was purchased from the American Type Culture Collection (ATCC, USA). DU145 cells were cultured in EMEM media (ATCC, 30‐2003, USA) supplemented with 10% fetal bovine serum (FBS, ThermoFisher, USA) and 1% penicillin/streptomycin (ThermoFisher, USA). Cells were maintained at 37°C in 5% CO_2_.

*Dosage information*: S‐methyl‐L‐cysteine sulfoxide (SMCSO) was purchased from LKT Laboratories, USA (CAS 6858‐87‐8, purity >98%). S‐methyl methanethiosulfonate (MMTSO) was purchased from Merck, Germany (CAS 2949‐92‐0, purity 97%). SMCSO and MMTSO were dissolved in water to create a 10 mM stock solution and diluted to the final treatment concentrations with water. Both were used in parallel with water (vehicle) controls. Both were made fresh on the day of cell treatments to avoid possible issues of compound stability.

*WST‐1 assay*: Briefly, cells were seeded at 1.25 × 10^5^ cells/mL in 96‐well plates in a final 200 µL/well volume. Cells were cultured under standard conditions for 24 h, after which media was removed, and cells were treated with SMCSO or MMTSO diluted in the medium and incubated for 24 h. Subsequently, ten microliters of WST‐1 reagent were added to each well and incubated in standard culture conditions. WST‐1 cleavage was measured after 30 min at 450 nm (sample) and 650 nm (background) using a FLUOstar Omega microplate reader (BMG Technologies, UK). Medium plus WST‐1 served as a blank, which was deducted from all values.

*Trypan blue cell viability assay*: DU145 cells were seeded at 2.5 × 10^5^ cells/mL in 12‐well plates in a final 1 mL/well volume and cultured under standard conditions for 48 h, after which cells were treated with SMCSO or MMTSO for 24 h. After 24 h, treatments were removed, all wells were washed with 1x PBS, and each well was trypsinized (Gibco, USA). Cells were incubated for 10 min after adding 500 µL of medium to each well. Cells were diluted 1:1 with trypan blue (ThermoFisher, USA). Images were taken, and cell viability was measured using a Countess II Automated Cell Counter (ThermoFisher, USA).

*Seahorse mitochondrial analysis*: DU145 cells were grown to 70%–80% confluency and plated at 3 × 10^4^ cells/well in a total volume of 200 µL in the Seahorse cell culture plates (Agilent, USA). Cells were cultured under standard conditions for 24 h before treatment to achieve a monolayer. The flux sensor cartridges were hydrated with 200 µL/well of XF Calibrant Solution (Agilent, USA) overnight at 37°C in a non‐CO_2_ incubator. After treatment, media was removed, and cells were washed and incubated with 180 µL of warmed assay medium: Agilent XF base DMEM, pH 7.4, supplemented with 2 mM L‐glutamine (Merck, Germany), 2 mM pyruvate (ThermoFisher, USA), and 10 mM glucose (ThermoFisher, USA) for 1 h in a 37°C non‐CO_2_ incubator. The flux sensor cartridges were loaded with the mitochondrial inhibitors purchased from Agilent, USA, according to the manufacturer's instructions. For the cell mito stress assay, oligomycin (1.0 µM), carbonyl cyanide‐4 (trifluoromethoxy) phenylhydrazone (FCCP, 0.5 µM), and rotenone/antimycin A (0.5 µM); for the real‐time ATP test, oligomycin (1.5 µM) and rotenone/antimycin A (0.5 µM); for the mito fuel flex test, UK5009 (2.0 µM), Etomoxir (4.0 µM), and BPTES (3.0 µM). Oxygen consumption rate (OCR) and extracellular acidification rate (ECAR) values were obtained, and metabolic parameters were derived from calculations according to the manufacturer's instructions. All data were normalized to either total protein concentration for XFp assays or signal‐to‐blank ratio (determined by Hoechst staining) for XFe96 assays.

*RNA extraction and bioinformatics*: Briefly, DU145 cells were seeded on 6‐well plates and were treated with the appropriate treatment or vehicle in cell culture medium either supplemented with 5.5 mM or 25 mM glucose. RNA was extracted using the RNeasy kit (Qiagen, Germany) and QIAzol (Qiagen, Germany) according to the manufacturer's instructions. RNA was sent to Macrogen Europe, Netherlands; quality and quantity were assessed by Macrogen before the samples were sent for TruSeq Stranded mRNA Library Preparation poly(A) selection sequencing, performed on an Illumina NovaSeq 2× using 150 base paired‐end reads generating 30 million paired‐end reads per sample. RNA was analyzed as described by Bernuzzi et al. [24]. Briefly, reads were pre‐processed using FASTQC (version 0.11.9) and FASTP (version 0.23.1), mapped to the human reference genome using HISAT2 (version 2.1.0), and further assembled into full‐length transcripts using StringTie (version 1.2.2). Differential expression analysis was used to generate the differentially expressed genes (DEGs) using R Studio (version 4.2.1) and DESeq2 (version 1.24.0). Functional analysis was performed on the DEGs using the Hallmark pathway.

*Metabolomic analysis*: DU145 cells were seeded in T75 flasks, and once 80% confluency was achieved, cells were treated with the appropriate treatment or vehicle in cell culture medium either supplemented with 5.5 or 25 mM glucose. Cell pellets and supernatants were collected from each flask and sent for untargeted global metabolomics analysis by Metabolon, USA. Samples were prepared using the automated MicroLab STAR system from Hamilton Company. The resulting extract was divided into five fractions: two for analysis by two separate reverse phase (RP)/UPLC‐MS/MS methods with positive ion mode electrospray ionization (ESI), one for analysis by RP/UPLC‐MS/MS with negative ion mode ESI, and one for analysis by HILIC/UPLC‐MS/MS with negative ion mode ESI. Several QCs and controls were analyzed in conjunction with the experimental samples. Raw data were extracted, peaks were identified, and QC processing was carried out using Metabolon's hardware and software. Peaks were quantified using area‐under‐the‐curve and allocated into several biologically relevant classes: amino acids, carbohydrates, vitamins, tricarboxylic acid cycle (energy), lipids, nucleotides, peptides, and xenobiotics. For functional analysis, the normalized raw LCMS/MS values with their corresponding Human Metabolome Database (HMDB) ID, Kyoto Encyclopedia of Genes and Genomes (KEGG) ID, or name itself were entered into the MetaboAnalyst 5.0 online platform.

*Amino acid extraction and quantification*: Cell fractions and supernatants were extracted using a buffer consisting of 50% methanol, 30% acetonitrile, and 20% MilliQ water before samples were placed into a vacuum centrifuge dryer for 30 min before resuspension with 90 µL of 60% acetonitrile in water. In separate vials, 90 µL of each sample was added alongside 10 µL of 2.5 mM stable isotope‐labeled amino acid mix (MSK‐CAA‐1, Cambridge Isotope Laboratories, UK). For the calibration curve, 17 amino acids in 0.1 M hydrochloric acid (79248‐5 × 2ML, Supelco, USA) were diluted in 60% acetonitrile in water to give a 2.5 mM stock. The stock was diluted further from 0 µM to the highest concentration of 1 mM in fivefold concentrations. Quantification of amino acids was carried out as previously described by Perez‐Moral et al. [25].

*Statistical analysis*: Statistical analysis was performed in GraphPad Prism, USA (version 10.1.2). Where appropriate, significance was evaluated using one‐way ANOVA followed by Tukey's honest statistical hypothesis test for multiple comparisons, unpaired two‐way *T*‐tests, hypergeometric comparison tests, or two‐way ANOVA followed by the Benjamini Hochberg correction, with a *p* < 0.05 indicating statistical significance. Data are expressed as mean ± standard deviation (SD) from three to five biological replicates.

## 3 Results

### 3.1 High Concentrations of MMTSO, but Not SMCSO, Reduces WST‐1 Activity and Cell Viability in DU145 Cells

**FIGURE 2:**
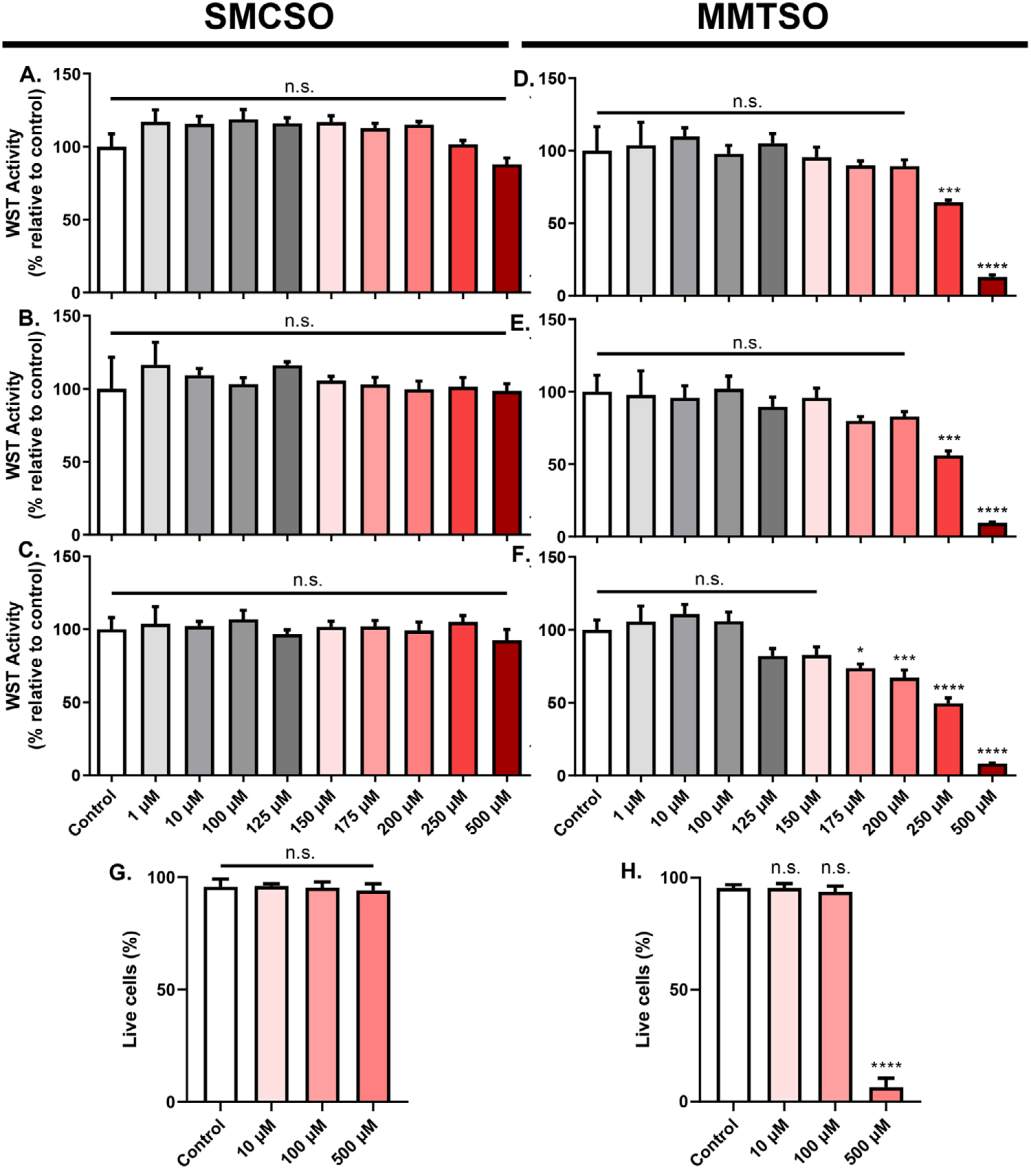
WST‐1 activity and cell viability assessment of DU145 cells following SMCSO and MMTSO treatments. DU145 were treated for 24 h with a range of concentrations of SMCSO or MMTSO in varying glucose concentrations basal (5.5 mM, A and D), intermediate (10 mM, B and E), and high glucose (25 mM, C and F) before the WST‐1 assay or trypan blue staining were conducted. (A–C) SMCSO did not affect WST‐1 activity in all glucose concentrations. (D–F) High concentrations of MMTSO altered WST‐1 activity in all glucose concentrations. (G) SMCSO did not affect percentage of live cells. (H) MMTSO reduced percentage of live cells only at 500 µM. Values represent mean ± SD. Statistical analysis was carried out with one‐way ANOVA with Tukey multiple comparisons for each time point. n.s., not significant; **p < 0.01; ***p < 0.001; ****p < 0.0001. Results are representative of *n* = 3–5 biological replicates.

We first determined the effects of SMCSO and MMTSO on WST‐1 activity and on cell viability (using the trypan blue assay). SMCSO did not affect WST‐1 activity in all glucose environments (Figure 2A–C). However, MMTSO concentrations above 250 µM decreased WST‐1 activity when supplemented in basal and intermediate glucose environments (Figure 2D–E). In contrast, concentrations higher than 175 µM reduced WST‐1 activity in the high glucose environment (Figure 2F). We then determined the percentage of live cells (cell viability) using the trypan blue assay and a Countess cell imager. Paralleling the result for WST‐1, SMCSO did not affect cell viability (Figure 2G). However, MMTSO treatment at 500 µM reduced cell viability by 90% (Figure 2H).

### 3.2 MMTSO Exposure Reduced Mitochondrial Metabolism in DU145 Cells; SMCSO Did Not

**FIGURE 3:**
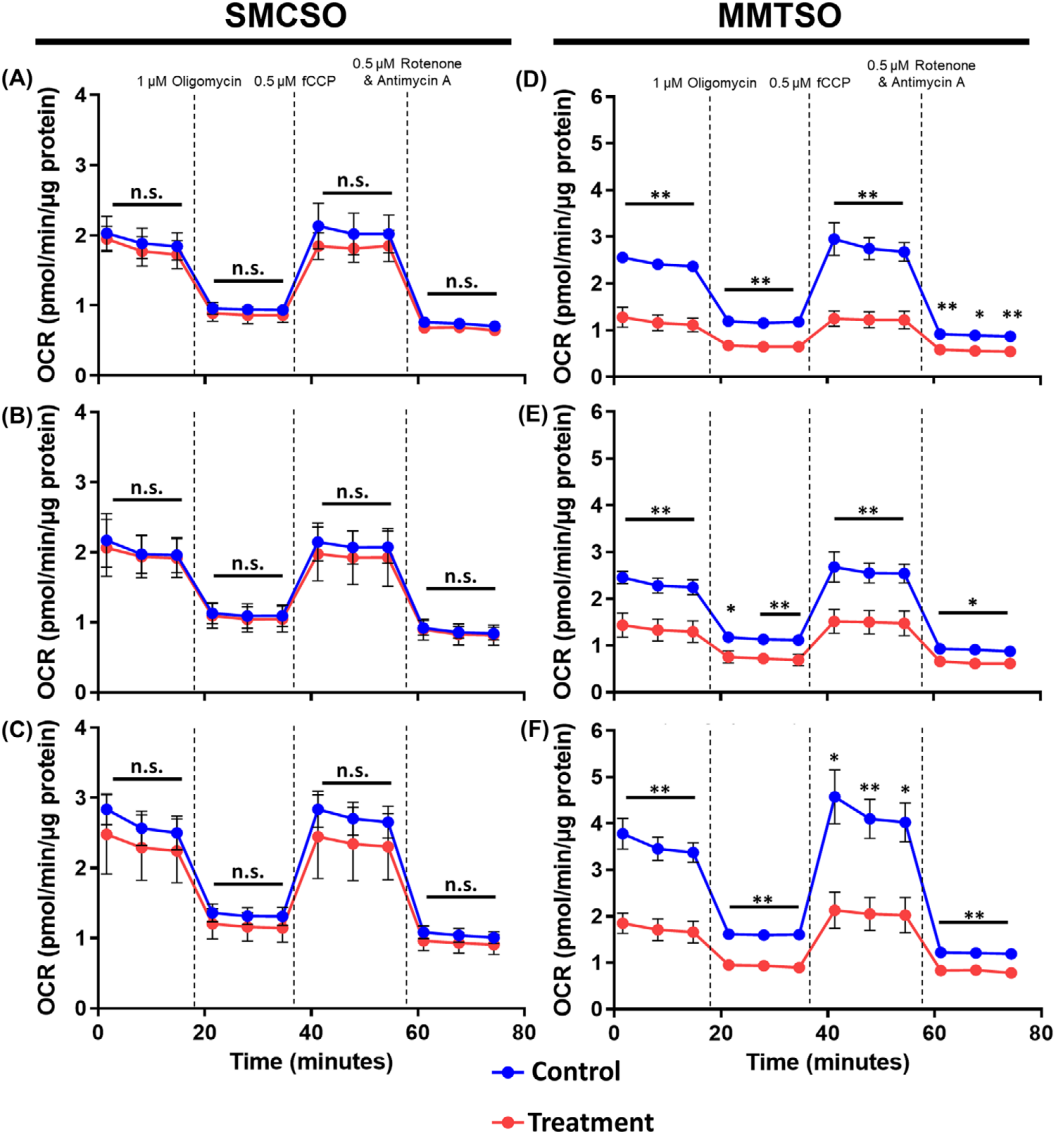
The effects of SMCSO and MMTSO on cellular respiration as measured by mitochondrial stress tests. DU145 cells were treated for 24 h with vehicle as a control or 100 µM SMCSO or 100 µM MMTSO under basal (5.5 mM, A and D), intermediate (10 mM, B and E), and high glucose (25 mM, C and F) environments before the cell mitochondrial stress test was carried out using the Seahorse XFp Bioanalyser. (A–C) SMCSO exposure for 24 h did not affect mitochondrial metabolism. (D–F) MMTSO exposure reduced mitochondrial metabolism. Values represent mean ± SD. Statistical analysis was carried out with unpaired two‐way *T*‐test for each time point, n.s., not significant; *p < 0.1; **p < 0.01. Results are representative of *n* = 3 biological replicates.

Next, we assessed whether SMCSO and MMTSO exposure affected the mitochondria activity in DU145 cells using the Seahorse XFp Bioanalyser. Compared to the control, SMCSO had no significant effect on mitochondrial metabolism (red lines in Figure 3A–C). MMTSO exposure at 100 µM for 24 h reduced mitochondrial metabolism (basal and maximal respiration) in all three glucose environments (Figure 3D–F). Overall, MMTSO, but not SMCSO, reduced mitochondrial metabolism of DU145 cells.

### 3.3 MMTSO Reduced Mitochondrial ATP and Oxidative Phosphorylation (%) Dose‐Dependently

**FIGURE 4:**
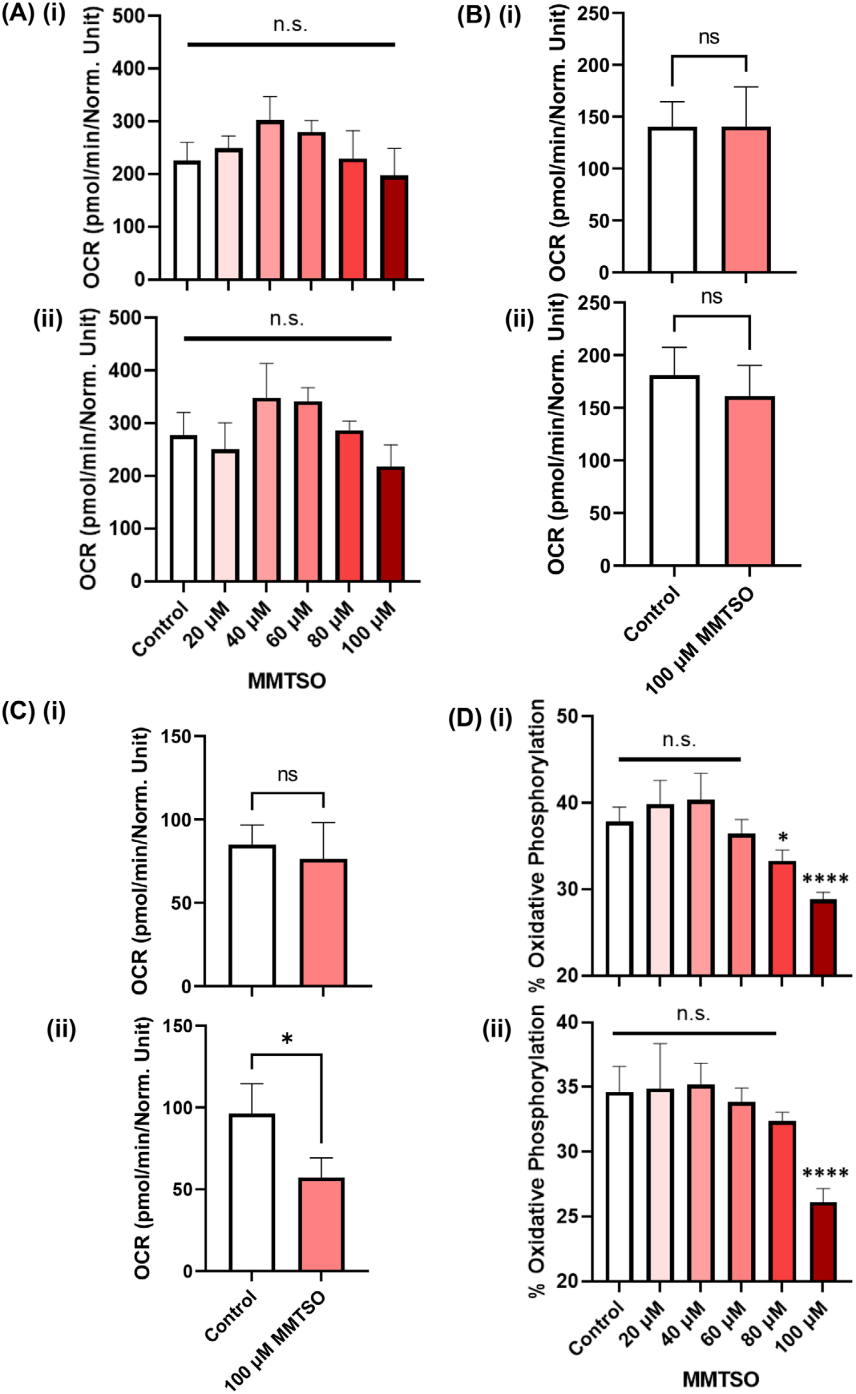
The effects of MMTSO on real‐time ATP production. DU145 cells were treated for 24 h with vehicle or MMTSO under basal (5.5 mM) and high (25 mM) glucose before the real‐time ATP test was used on the Seahorse XFe96 Bioanalyser. Treatment with 100 µM MMTSO reduced both mitochondrial ATP production and % oxidative phosphorylation compared to control. (A) MMTSO did not affect total ATP in (i) basal and (ii) high glucose, (B) MMTSO did not affect glycolytic ATP in (i) basal and (ii) high glucose, (C) MMTSO does not affect mitochondrial ATP in (i) basal, (C) MMTSO reduced mitochondrial ATP in (ii) high glucose, (D) MMTSO reduced % oxidative phosphorylation in (i) basal and (ii) high glucose. Values represent mean ± SD. Statistical analysis was carried out with either one‐way ANOVA followed by Tukey's honest statistical hypothesis test for multiple comparisons (A and D), or unpaired two‐way *T*‐test (B and C) for each time point. n.s., not significant; *p < 0.1; ****p < 0.0001. Results are representative of *n* = 3–5 biological replicates.

To further consider the effect of MMTSO on prostate metabolism, total ATP production (sum of glycolytic and mitochondrial ATP) and oxidative phosphorylation (%) were assessed using the Seahorse XFe96 Bioanalyser. Although MMTSO had no significant effect on total ATP concentrations (Figure 4A) and glycolytic ATP at 100 µM MMTSO in basal and high glucose (Figure 4B), there was a significant reduction in mitochondrial ATP at 100 µM MMTSO in the high glucose environment (Figure 4C(ii)). MMTSO also reduced the percentage of oxidative phosphorylation dose‐dependently in basal and high glucose (Figure 4D) [23].

### 3.4 MMTSO Affected Genes and Pathways Involved in Energy Metabolism, Antioxidant Response, Apoptosis, and Immune Function

**FIGURE 5:**
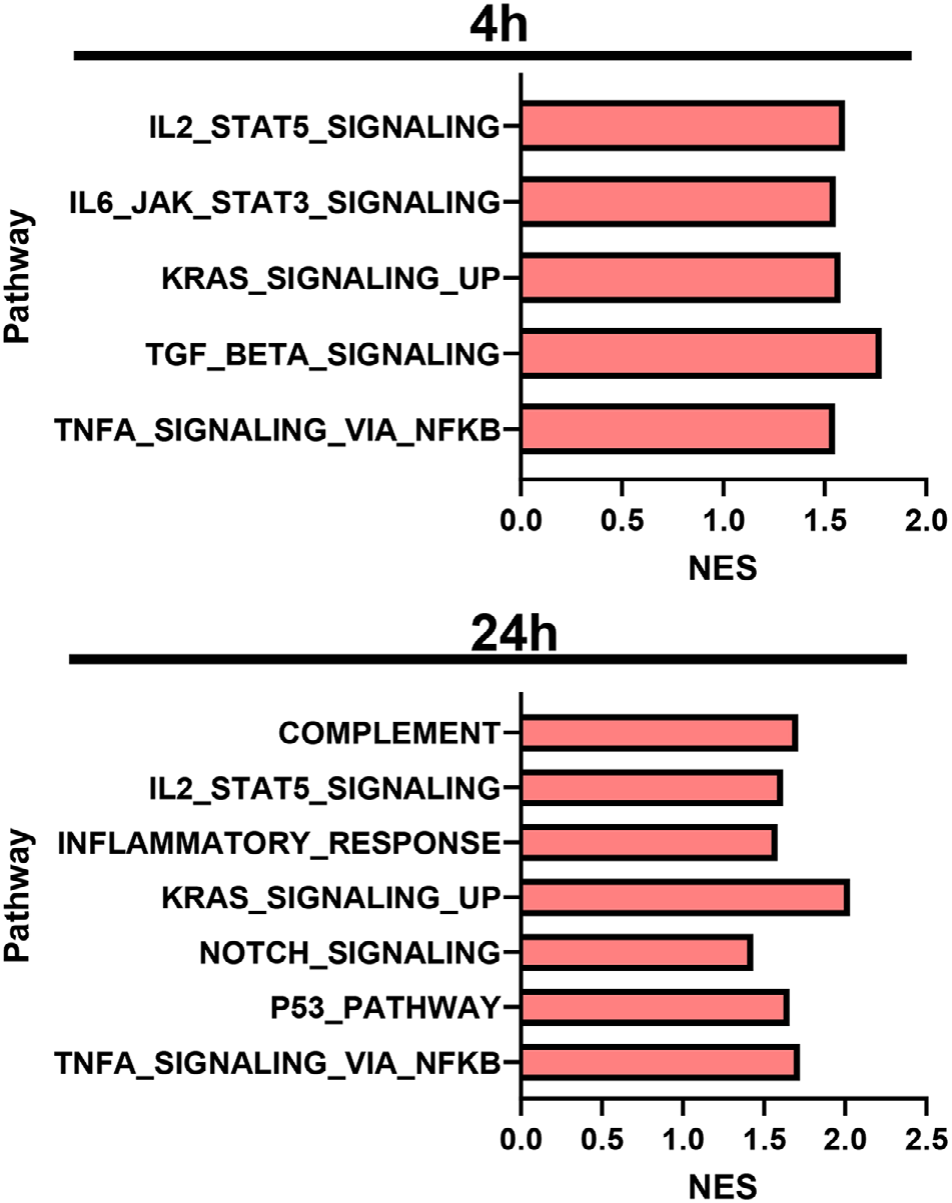
Pathway analysis using the Hallmark database in response to MMTSO exposure to DU145 cells at 4‐h and 24‐h in basal glucose. Pathway analysis highlighted key immune function and tumor‐associated pathways were altered due to MMTSO exposure. The *x*‐axis represents the normalized enrichment score (NES); *y*‐axis represents pathway. The NES is produced from the GSEA and denotes the significance of the pathway normalized to the size of each pathway. A positive NES represents an increase in the pathway; a negative NES represents a decrease in the pathway. IL2, interleukin 2; IL6, interleukin 6; JAK, Janus kinase; KRAS UP, genes upregulated by KRAS signaling; P53, tumor protein p53; STAT3 or 5, signal transducer and activator of transcription 3 or 5; TGF, transforming growth factor; TNFA, tumor necrosis factor alpha. Statistical analysis was carried out with two‐way ANOVA followed by Benjamini‐Hochberg correction for each time point, and significant to *q* value ≤ 0.05. Results are for *n* = 5 biological replicates.

To understand the mechanisms involved in reducing mitochondrial ATP by MMTSO, we assessed the transcriptomic profile of DU145 cells exposed to SMCSO and MMTSO. We further carried out gene set enrichment analysis (GSEA) using the Hallmark database to assess how each treatment influenced the profiles of gene sets within biological pathways. A comparison of differentially expressed genes highlighted critical genes related to energy and mitochondrial metabolism (Table 1), antioxidant response (Table 2), and apoptosis (Table 3), being significantly affected by MMTSO exposure in basal glucose and enhanced further in high glucose. Both 4‐h and 24‐h treatment with MMTSO led to the upregulation of key pathways relating to immune function, with an increase in genes, including interleukin‐signal transducer and activator of transcription 5 (IL2‐STAT5) (Figure 5). Interestingly, 24‐h MMTSO exposure upregulated tumor‐associate pathway P53 (Figure 5). Transcriptomic data were also obtained for SMCSO treatments, but these cause no significant changes in relative gene transcript levels (data not shown).

**TABLE 1:** Comparison of differentially expressed genes involved in energy and mitochondrial metabolism in response to treatment with MMTSO.

		Basal glucose		High glucose	
Gene symbol	Gene	Log_2_ fold change^a^	*q* value^b^	Log_2_ fold change^a^	*q* value^b^
***CPT1A***	Carnitine palmitoyltransferase 1A	0.32	< 0.001	0.35	0.004
***ENO2***	Enolase 2	0.29	< 0.001	0.35	< 0.001
***GALK1***	Galactokinase 1	0.14	0.05	0.37	< 0.001
***HK1***	Hexokinase 1	0.13	< 0.001	0.13	< 0.001
***NSDHL***	NAD(P) dependent steroid dehydrogenase‐like	0.26	< 0.001	0.25	< 0.001
***PALD1***	Phosphatase domain containing paladin 1	0.23	< 0.001	0.22	< 0.001
***SDHA***	Succinate dehydrogenase complex flavoprotein subunit A	0.01	0.82	0.07	0.002
***SDHB***	Succinate dehydrogenase complex flavoprotein subunit B	0.06	0.18	0.14	< 0.001

^a^ Log_2_ fold change of MMTSO treated cells compared to control.

^b^ *q* values were determined through two‐way ANOVA followed by Benjamini‐Hochberg correction.

**TABLE 2:** Comparison of differentially expressed genes involved in antioxidant responses in response to MMTSO.

		Basal glucose		High glucose	
Gene symbol	Gene	Log_2_ fold change^a^	*q* value^b^	Log_2_ fold change^a^	*q* value^b^
***GCLC***	Glutamate‐cysteine ligase catalytic subunit	−0.18	< 0.001	−0.43	0.004
***GPX8***	Glutathione peroxidase 8	−0.21	< 0.001	−0.48	< 0.001
***GSR***	Glutathione‐disulfide reductase	−0.25	< 0.001	−0.45	< 0.001
***NQO1***	NAD(P)H quinone dehydrogenase 1	−0.21	< 0.001	−0.33	< 0.001
***SLC6A9***	Sodium‐ and chloride‐dependent glycine transporter 1	−0.52	< 0.001	−1.00	< 0.001
***SRXN1***	Sulfiredoxin 1	−0.32	< 0.001	−0.52	< 0.001

^a^ Log_2_ fold change of MMTSO treated cells compared to control.

^b^ *q* values were determined through two‐way ANOVA followed by Benjamini–Hochberg correction.

**TABLE 3:** Comparison of differentially expressed genes involved in apoptosis in response to MMTSO.

		Basal glucose		High glucose	
Gene symbol	Gene	Log_2_ fold change^a^	*q* value^b^	Log_2_ fold change^a^	*q* value^b^
***BAK1***	BCL2 antagonist/killer 1	0.20	< 0.001	0.25	< 0.001
***CARD19***	Caspase recruitment domain family member 19	0.22	0.002	0.22	< 0.001
***FADD***	Fas associated via death domain	0.11	0.02	0.22	< 0.001
***TNFAIP8***	TNF alpha induced protein 8	0.30	< 0.001	0.19	0.003

^a^ Log_2_ fold change of MMTSO treated cells compared to control.

^b^ *q* values were determined through two‐way ANOVA followed by Benjamini‐Hochberg correction.

### 3.5 MMTSO Increased Fatty Acid Dependency and Caused Metabolite Changes in Central Cellular Metabolism Pathways, Including the Tricarboxylic Acid (TCA) Cycle and Amino Acids Metabolism

**FIGURE 6:**
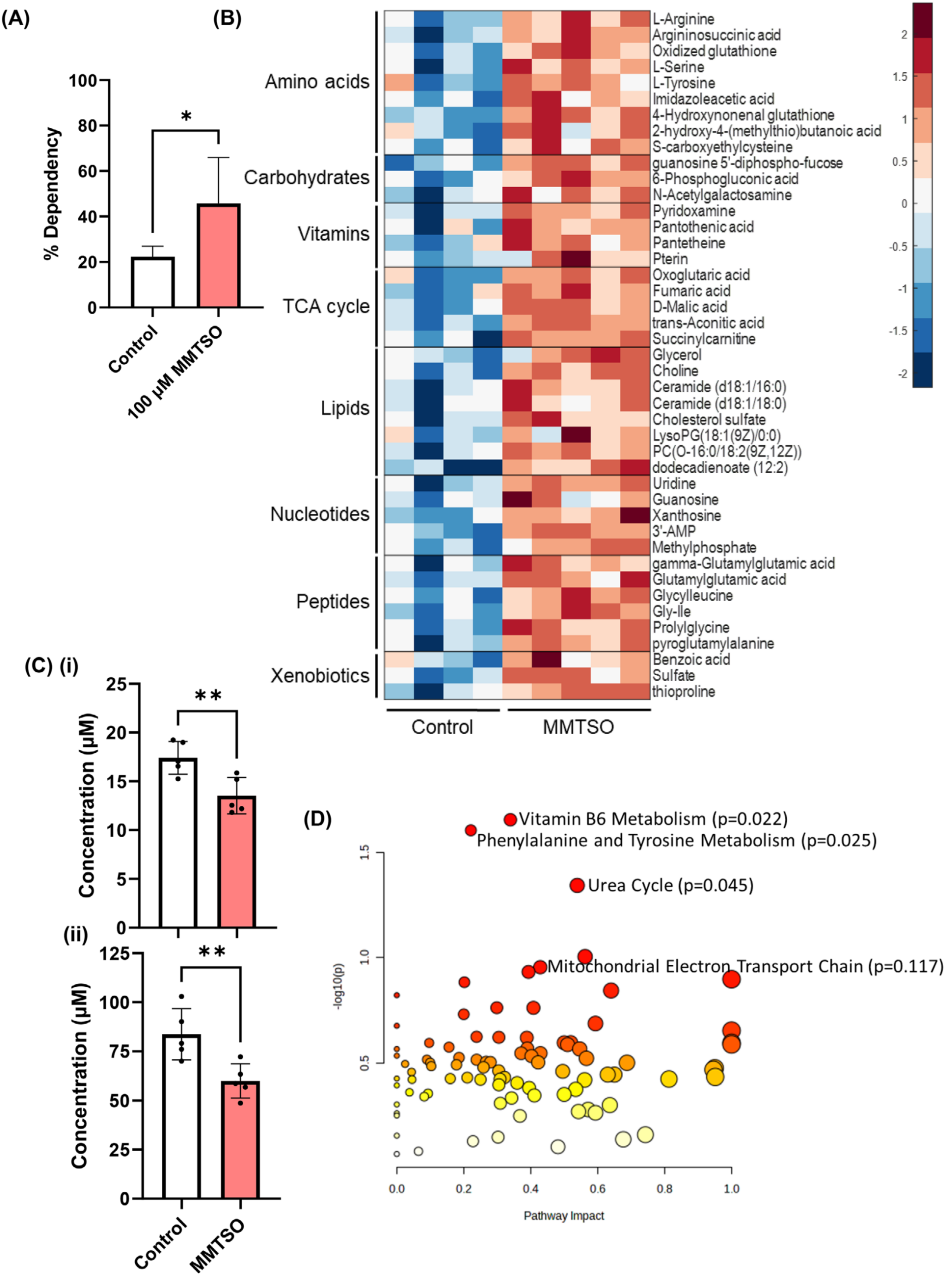
Fatty acid dependency and metabolite profile analysis following MMTSO exposure to DU145 cells. (A) % dependency of fatty acids. MMTSO (100 µM), compared to control, led to increased fatty acid dependency using the mito fuel flex test. (B) Heatmap analysis of significantly different metabolites (*p* < 0.05) detected by Metabolon within cells treated with 100 µM MMTSO with respect to control in high glucose. Plots were generated using z‐scores using MatLab; metabolite change is scaled from −2 to 2 using a color scale of red (decreased) to blue (increased) with no color representing no change. There were increased compounds following MMTSO treatment compared to control, highlighting increased compounds in the tricarboxylic acid (TCA) cycle biological class. (C) Amino acid analysis following 100 µM MMTSO exposure in high glucose in cell samples. The concentration (µM) of each was plotted. After 24‐h treatment with control and MMTSO, there was decreased (i) glycine and (ii) glutamate. (D) Pathway analysis metabolome view from DU145 cell exposure to 100 µM MMTSO in high glucose using Metaboanalyst. *Y*‐axis values are presented as −log10(*p*) for graphical separation and X‐axis values for pathway impact. The match status and centrality within pathways confer higher pathway impact. Vitamin B6 metabolism, phenylalanine and tyrosine metabolism and urea cycle significantly differed (*p* < 0.05) between control and MMTSO treated samples. Although not significant, the mitochondrial electron transport chain is also highlighted (*p* = 0.117). Values represent mean ± SD. Statistical analysis was carried out with either unpaired two‐way *T*‐test (A and C), hypergeometric comparison tests (B), or one‐way ANOVA followed by Tukey's honest statistical hypothesis test for multiple comparisons (D), or for each time point. n.s., not significant; *p < 0.1; **p < 0.01. Results are representative of *n* = 3–5 biological replicates.

As the transcriptomics revealed that MMTSO treatment of DU145 cells upregulated the expression of CPT1a, a crucial gene regulating fatty acid metabolism (Table 1), the mito fuel flex test was used to determine the effect of MMTSO on fatty acid metabolism. There was an increased need for fatty acid metabolism to maintain baseline respiration following MMTSO treatment (Figure 6A). Global metabolite analysis following untargeted metabolite analysis indicated that MMTSO treatment increased key tricarboxylic acid metabolites, amino acids, urea cycle intermediates, purines and pyrimidines, and peptides and lipids (Figure 6B). Particularly, there was a significant decrease in glycine and glutamate (Figure 6C). Pathway analysis using the Metaboanalyst online platform indicated that the exposure of MMTSO to DU145 cells in high glucose led to significant alterations in vitamin B6 metabolism (*p* = 0.022), phenylalanine and tyrosine metabolism (*p* = 0.025), and the urea cycle (*p* = 0.045), Figure 6D. Interestingly, the mitochondrial electron transport chain was ranked 6 out of 15, although not significant (*p* = 0.117).

## 4 Discussion

Epidemiological studies strongly support the beneficial properties of consuming cruciferous and alliaceous vegetables on human health [16, 26, 27, 28, 29, 30]. The possible effects of the sulfur‐containing compound SMCSO in these vegetables have remained largely unknown. This study investigated the metabolic effects of SMCSO and its human metabolite, MMTSO, on prostate cancer metabolism. Here, we report the first account that MMTSO altered WST‐1 activity and cell viability at high concentrations (but SMCSO did not), and MMTSO dose‐dependently reduced mitochondrial metabolism, mitochondrial ATP production, and oxidative phosphorylation and, in parallel, altered genes and pathways involved in cellular energy metabolism and immune response and the tricarboxylic acid (TCA) cycle. Overall, these data are consistent with MMTSO exerting metabolic effects on the prostate cancer cells. It is worth noting that the observed significant effects of MMTSO on mitochondrial metabolism in the DU145 cells were not associated with changes in cell viability.

Mitochondria play a role in the metabolic ability and proliferation of prostate cancer [31, 32]. Lower OCRs have been reported in human liver cancer cells (HepG2) compared to non‐cancerous controls [11]. The evidence observed here with SMCSO agrees with a previous study using a prostate cancer cell line where SMCSO had no significant effect on mitochondrial function [10]. Here, we show that MMTSO reduced mitochondrial ATP production and oxidative phosphorylation, suggesting reduced metabolic capacity of the DU145 prostate cancer cells. Thiosulfonic compounds (like MMTSO) have been shown to bind strongly and selectively to the STAT3‐SH2 domain and to cause modest antiproliferative and cytotoxic effects on the colon cancer cell line, HCT‐116 [33]. The effects of MMTSO reported here may be a consequence of direct interactions between MMTSO and STAT3‐SH2, which has been associated with mitochondrial pyruvate metabolism and activation of target genes [33, 34]. This leads to reduced mitochondrial function, cell proliferation, and increased apoptosis. As there is limited evidence of the biological activity of SMCSO and MMTSO, with much of it being focused on antidiabetic effects in rodents [17, 18, 19, 35], the present work is the first evidence that MMTSO exposure drives a shift in mitochondrial metabolism in DU145 prostate cancer cells.

Like other cancers, prostate cancer can develop immune mechanisms to support evasion from specific immune responses and regulation in the body [36]. Here, we show that MMTSO increased the upregulation of the IL‐2‐STAT5 gene set and other immune‐related pathways. These mechanisms include modifying the tumor microenvironment to a more immunosuppressive environment, rendering T‐cell responses ineffective, and reducing antigen presentation [37]. Interestingly, a human study demonstrated that patients with prostate cancer treated with a standard treatment of zoledronate (medication to reduce calcium loss from bones due to cancer) and IL‐2 for 12 months had significantly increased survival rates compared to those just treated with zoledronate alone [38]. Further, sulfur metabolites, including allicin with a similar chemical structure to MMTSO, have been shown to cause upregulation of the IL‐2 immune regulation pathway [39]. Similar upregulation is observed following MMTSO treatment, which may relate to the presence of thiol groups in both MMTSO and allicin [9]. The observation that MMTSO causes an upregulation in IL‐2 signaling gene sets provides evidence that MMTSO could beneficially affect immunomodulatory processes within the context of prostate cancer and warrants further investigation.

The antioxidant system comprises various enzymes, including those involved in glutathione metabolism (GCLC and GSR) and phase II detoxifying enzymes such as (NQO1), which play a crucial role in ROS regulation and protecting cells from damage [40, 41]. GCLC (involved in glutathione biosynthesis from glutamate), GSR (involved in the conversion of oxidized glutathione to reduced glutathione), and NQO1 (involved in the reduction of quinones to hydroquinones) have all been reported to be overexpressed in many types of cancer, including renal, lung, and prostate cancers [42, 43]. The observation that MMTSO caused downregulation of GCLC, GSR, and NQO1 genes provides evidence that MMTSO could play a role in oxidative stress and antioxidant defense of prostate cancer. Inhibition of NQO1 decreased ROS and p53 levels and suppressed NF‐_K_B interaction, modulating the inflammatory response associated with prostate cancer [44].

The extrinsic apoptotic pathway is initiated by the binding of ligands to cell surface death receptors linked with the Fas‐associated death domain (FADD) [45]. Upregulation of FADD was shown following MMTSO treatment, suggesting increased apoptosis. BCL‐2 antagonist killer (BAK1) is a serine/threonine kinase that acts as a pro‐apoptotic regulator in the intrinsic apoptotic pathway [46, 47]. We show that BAK1 was upregulated following MMTSO treatment, promoting apoptosis. Thiosulfinates isolated from the garlic chive, including MMTSI, were reported to have promising inhibitory effects on the proliferation of human prostate and colon cancer cell lines through both caspase‐dependent and caspase‐independent apoptosis pathways [48, 49, 50]. Interestingly, allicin, another organosulfur compound with a similar structure to MMTSO, inhibited MMP‐2 and MMP‐9 expression, leading to inhibition of cell proliferation and induction of apoptosis [51]. These studies provide evidence that similar compounds to MMTSO act on pathways related to cell proliferation and apoptosis, suggesting MMTSO could also have similar properties.

The prostate has a unique metabolic profile compared to other organs. Prostate epithelial secretory cells are highly glycolytic, secrete citrate, and have inactive oxidative phosphorylation and TCA cycles [52]. However, in malignant transformation to prostate cancer, the cells alter their central metabolism to meet the high energy demands of cancer development, increasing oxidative phosphorylation [53]. In this context, it seemed plausible to suggest that MMTSO increased flux through the TCA cycle to reduce lipid synthesis and histone acetylation, which are risk factors and may interact with cancerous cells in a way that affects the metabolic capacity of the prostate cancer cell [54]. While a recent study showed that supplementation of varying doses of SMCSO did not alleviate metabolic syndrome in mice fed a high‐fat diet [55], our study showed that MMTSO influenced lipid metabolism, highlighting that MMTSO rather than SMCSO is the critical metabolite underpinning metabolic effects and that future in vitro/vivo studies should be carried out using MMTSO.

Bernuzzi et al. recently demonstrated that another sulfur compound, sulforaphane, can interfere with amino acid metabolism by inhibiting serine biosynthesis, reducing glucose flux towards the serine synthetic pathway, and redirecting glycine and glutamate towards the glutathione pool [24]. To our surprise, our research showed MMTSO increased amino acids such as tyrosine, phenylalanine, serine, urea cycle intermediates (arginine and argininosuccinic acid), and nucleotides such as purines (guanosine and xanthosine) and pyrimidines (uridine), suggesting a shift in metabolism. We postulate that the additional serine drives increased folate cycle activity, where the methyl group is used to generate purines. In contrast, tyrosine is broken into fumarate, which feeds into the TCA cycle and is converted to aspartate, which is then combined with carbamoyl phosphate to be redirected for pyrimidine synthesis. Cancer cells, due to their high proliferative rates, have nucleotide pool imbalances, resulting in replication stress and impaired DNA damage repair, which ultimately results in DNA damage [56]; as a result, the ability of MMTSO to increase both purines and pyrimidines at the same time may restore nucleotide balance, thereby maintaining effective DNA repair mechanisms and thus preventing DNA damage [57].

A key consideration when planning experiments to test the possible effects of the abundant sulfur‐containing broccoli metabolite SMCSO and its major human metabolite MMTSO is the physiologically relevant concentrations to use as treatments. A previous dietary intervention involving the consumption of broccoli soups reported the average concentration of SMCSO in urine to be ∼100 µM [58], and we report here that this concentration did not negatively affect WST‐1 activity or cell viability of cultured prostate cancer cells. Regarding MMTSO, no reliable method for quantification has been published, and there are no reports of concentrations of MMTSO in urine, plasma, or tissue samples. Thus, the physiologically relevant concentration of MMTSO is not known. However, urinary concentrations of SMCSO have been reported up to around 100 µM, and prostate biopsy concentrations were reported up to around 15 nmoles/g fresh tissue [16]. If it is assumed that 1 kg of prostate tissue has a volume of approximately 1 L, the reported tissue concentration of 15 nmoles/g fresh tissue is around 15 µM (intracellular concentration). When we treat the prostate cells, we are treating them extracellularly (as urinary reflux of the prostate tissue would achieve), and so we considered it physiologically relevant to treat them at the 100 µM concentration, and the same concentrations were used for SMCSO. We reported that this MMTSO dose did not negatively affect WST‐1 activity or cell viability, which allowed for proof‐of‐principle analysis of the effects of this metabolite on prostate cancer cells.

To conclude, we have shown that the sulfur‐containing compound MMTSO, but not SMCSO, significantly reduced mitochondrial metabolism, mitochondrial ATP generation, and oxidative phosphorylation in DU145 prostate cancer cells, with the strongest effects observed in high glucose environments. MMTSO exposure influenced key immune signaling pathways and mitochondrial regulatory genes, altered vital metabolic pathways in cellular metabolism, and increased fatty acid dependency. Overall, these data show that MMTSO alters several features of energy metabolism in DU145 prostate cancer cells. This study adds to the current understanding of the biological activity of MMTSO on the prostate. It gives insight into mechanistic and metabolic links to prostate cancer cell metabolism that could contribute to the health benefits associated with a broccoli‐rich diet. Further dietary human studies are warranted to test this further with a product rich or pure in SMCSO to test its effect in vivo on prostate cancer metabolism.

## Conflicts of Interest

RFM and MHT are inventors of patents concerned with the development and use of high glucoraphanin broccoli. No potential conflict of interest was reported by the other authors.

## Data Availability

The data that support the findings are available on request from the corresponding author.
